# APOE4 expression confers a mild, persistent reduction in neurovascular function in the visual cortex and hippocampus of awake mice

**DOI:** 10.1177/0271678X231172842

**Published:** 2023-06-23

**Authors:** Orla Bonnar, Kira Shaw, Silvia Anderle, Dori M Grijseels, Devin Clarke, Laura Bell, Sarah L King, Catherine N Hall

**Affiliations:** School of Psychology and Sussex Neuroscience, 1948 University of Sussex, Falmer, Brighton, East Sussex, UK

**Keywords:** Apolipoprotein E, neurovascular coupling, Alzheimer’s disease, two-photon microscopy, vasomotion

## Abstract

Vascular factors are known to be early and important players in Alzheimer’s disease (AD) development, however the role of the ε4 allele of the Apolipoprotein (APOE) gene (a risk factor for developing AD) remains unclear. APOE4 genotype is associated with early and severe neocortical vascular deficits in anaesthetised mice, but in humans, vascular and cognitive dysfunction are focused on the hippocampal formation and appear later. How APOE4 might interact with the vasculature to confer AD risk during the preclinical phase represents a gap in existing knowledge. To avoid potential confounds of anaesthesia and to explore regions most relevant for human disease, we studied the visual cortex and hippocampus of awake APOE3 and APOE4-TR mice using 2-photon microscopy of neurons and blood vessels. We found mild vascular deficits: vascular density and functional hyperaemia were unaffected in APOE4 mice, and neuronal or vascular function did not decrease up to late middle-age. Instead, vascular responsiveness was lower, arteriole vasomotion was reduced and neuronal calcium signals during visual stimulation were increased. This suggests that, alone, APOE4 expression is not catastrophic but stably alters neurovascular physiology. We suggest this state makes APOE4 carriers more sensitive to subsequent insults such as injury or beta amyloid accumulation.

## Introduction

The brain’s dense network of vasculature performs multiple roles to support neuronal health and function. For example, neurovascular coupling matches neuronal energy demands with the delivery of oxygen and glucose via the blood^1^ and vasomotion – slow (∼0.1 Hz) oscillations of arterioles^2,3^ – drives the clearance of waste products along blood vessels. In Alzheimer’s disease (AD) alterations in cerebral blood flow (CBF) control likely establish a vicious cycle that exacerbates pathology, whereby decreased blood flow increases Aβ accumulation by promoting its formation in hypoxic conditions. Blood flow is then limited by accumulating Aβ, as blood vessels are constricted by soluble Aβ, and accumulation of Aβ within vessels likely inhibits vasomotion.^2,4–7^ Vascular dysregulation occurs early in AD,^8^ so disrupted blood flow may be the primary cause of emerging AD pathology.

Recent work from our group has demonstrated heterogeneity in vascular function across brain regions, where blood supply was compromised in CA1 region of the hippocampus compared to primary visual cortex.^9^ Weaker neurovascular function in the hippocampus, and its vulnerability to hypoxia,^10^ are congruent with regional susceptibility in AD, where early in the disease sensory cortices are relatively spared compared to the hippocampus.

Whilst most AD cases are sporadic, the risk of developing the disease is increased 9–15 fold by the possession of an ε4 allele of the apolipoprotein E (APOE) gene.^11,12^ ApoE is involved in lipid transportation and is produced mainly by astrocytes, but also by vascular mural cells and microglia, and neurons under stress conditions.^12^ APOE4 expression exacerbates AD pathological features, increasing Aβ accumulation and tau phosphorylation and altering synaptic function.^13^ It also decreases blood brain barrier (BBB) integrity and causes pericyte damage in human and animal subjects.^14,15^ Additionally, anaesthetised APOE4 mice had reduced baseline and stimulus-induced CBF.^14,16^

As CBF changes occur before other AD pathology in humans,^8^ APOE4-mediated vascular dysfunction could be an important contributor to the earlier emergence of AD in APOE4 carriers. However, gaps in the data complicate our understanding of how APOE4 genotype could be promoting AD.

Firstly, neuronal activity has largely not been studied concurrently with vascular function, and reports as to the impact of APOE4 genotype on neuronal activity are variable. Decreases in neuronal activity have been reported in young APOE4 mice,^14^ and neuronal hyperactivity in old APOE4 mice.^17^ The potential direction of causality is unclear: could decreases in CBF be due to reduced neuronal drive, or could CBF be restricting neuronal function?

Secondly, the impact of APOE4 on hippocampal neurovascular coupling has not yet been studied, despite it being more sensitive to AD damage,^18^ a site of BBB breakdown in old APOE4 carriers,^15^ and having weaker neurovascular function than neocortical regions.^9^

Finally, animal studies in which CBF and functional hyperaemia are dramatically decreased in young mice ^14,16^ do not recapitulate the human condition in which young APOE4 carriers show, if anything, increased CBF compared to APOE3 carriers from young into early old age.^19,20^ BBB changes in human APOE4 carriers have also only been reported from old age,^15,21^ whereas these changes were observed in very young mice.^14^ It is therefore hard to interpret the relevance of rodent studies that show much more extreme changes in young animals ^14,16^ than are seen in humans.

An explanation for the greater impact of APOE4 in rodent studies than humans may be that carrying APOE4 increases the impact of stressors such as anaesthesia.^22,23^ Inhalation anaesthetics such as isoflurane seem to have a particularly large effect, reducing cognitive function acutely after surgery, before recovering to baseline levels after 10 days.^24^

To aid our understanding of how APOE4 impacts on cerebrovascular function, we therefore studied awake mice expressing human APOE3 or 4 in place of murine APOE, to avoid the potentially confounding effect of acute surgery and anaesthesia. We recorded neurovascular function up to 4 months after mice recovered from surgery (from young to late-middle age). We used 2-photon imaging to measure neuronal and vascular activity at a single vessel and neuronal level, as well as measuring net neuronal metabolism and vasodilation using haemoglobin spectrometry and laser doppler flowmetry. Finally, we studied both the primary visual cortex (V1) and the CA1 subfield of the hippocampus to probe whether APOE4 effects were more pronounced in the hippocampus.

We observed several subtle alterations in neurovascular function that persisted from young to late-middle age, including impaired vasomotion, neuronal hyperactivity and less reliable stimulus-evoked vascular responses. These data are consistent with the pattern of results observed in humans, and suggest APOE4 genotype does not directly catastrophically impair vascular function, but rather more subtly shifts neurovascular physiology such that it is more susceptible to further insults (e.g. surgery, ageing or beta amyloid accumulation).

## Materials and methods

Further details on materials and methods can be found in supplementary material.

## Animals

All experimental procedures were approved by the UK Home Office, in accordance with the 1986 Animal (Scientific Procedures) Act and reporting of this study was in compliance with the ARRIVE guidelines. Experiments were carried out on male and female homozygous APOE3-TR or APOE4-TR mice,^25,26^ crossed with animals expressing DsRed under the control of the NG2 promoter ^27^ or with animals expressing GCaMP6f under the control of the Thy1 promoter. APOE-TR mice were bred from an in-house colony (derived from founders provided by N. Maeda (UNC School of Medicine, USA)). All mice were on a C57BL/6 background and between 3–4 months old, unless otherwise specified. In ageing experiments, 6–7 month mice were aged from the 3–4 months cohort, while 12–13 month mice were a separate cohort. Animals were housed in a temperature-controlled room with a 12-hour light/dark cycle and free access to food and water. Animals had access to a running wheel, nesting material and mixed seeds (in addition to standard chow) and were largely singly housed. A moderately enriched environment was employed to minimise sensory deprivation, mitigate social isolation and thereby to better recapitulate the non-deprived state of most human APOE carriers.

Experimenters were not blinded but data analysis was automated to remove any subjectivity.

Animals that did not recover well from surgery were humanely sacrificed (V1:5 animals, HC: 4 animals), as were animals with poor quality cranial windows (V1: 0 animals, HC: 4 animals) or with no GCaMP6f expression when expected (V1: 1 animal, HC: 0 animals). In ageing experiments, for some animals the quality of the window deteriorated between the first and second time point and thus these were excluded from further experiments (V1: 3 animals). Furthermore, some animals were entered only into the early time point (V1: 9) and were intentionally sacrificed before the middle time point. Finally, during initial experiments some animals (V1:2) were imaged outside of the early time window (3–4 months) and thus only contributed to the middle age group.

## Surgical preparation

Animals were surgically implanted with a cranial window over the primary visual cortex or the CA1 subfield of the hippocampus as previously described.^9^

## In vivo experiments

For all in vivo experiments, animals were head-fixed atop a cylindrical treadmill and locomotion was recorded using a rotary encoder (Kuebler). Recordings were made during ‘baseline’ conditions (in the dark), and in animals with a window over V1, during visual stimulation. A drifting grating (315°, 2 Hz full screen stimulus, with alternating spatial frequency trials of either 0.04 or 0.2 cycles per degree) was presented for 5 seconds (Asus, screens ∼17 cm from mouse), with a 20 second interval.

### 2-Photon microscopy

Animals were imaged using a 2-photon microscope (Scientifica) and a mode-locked Ti-sapphire laser (Coherent). Images were acquired using a 20× (XLUMPlanFL N, Olympus) or 16× (LWD, Nikon) water immersion objective from tissue excited with a laser wavelength of 940 nm or 970 nm. The objective was shielded with tape to minimise light artefacts from visual stimulation. Images were collected using SciScan (Scientifica) software.

Prior to imaging, animals were injected with a fluorescent dextran. Those with green fluorescence (i.e. GCaMP6f) were injected with 100 µL 2.5% Texas red (Sigma-Aldrich) either subcutaneously (3 kDa) or intravenously via the tail vein (70 kDa), and those with red fluorescence (i.e., DsRed in vascular mural cells) were injected intravenously with 2.5% fluorescein isothiocyanate-dextran (70 kDa FITC-dextran; Sigma-Aldrich), allowing for visualisation of the vasculature.

### Oxy-CBF probe

Net (∼500 µm^2)^ haemodynamic measurements were recorded using a combined laser Doppler flowmetry/haemoglobin spectroscopy probe (Oxy-CBF probe; Moor Instruments^28^) at an acquisition rate of 40 Hz. Measures of total haemoglobin (HbT), blood flow (flux) and oxygen saturation (sO_2_) and cerebral metabolic rate of oxygen consumption (CMRO_2_ – calculated using equation (i) below) were obtained both at baseline, and in V1, during visual stimulation.

(i) $${\mathrm{CMRO}}_{2}=\mathrm{CBF}(t)\times \frac{\mathrm{HbR}\;(t)}{\mathrm{HbT}(t)}$$

CBF represents cerebral blood flow, t represents time, HbR and HbT represent deoxygenated and total haemoglobin respectively.

## In vivo data analysis

### Vascular diameter and RBCV measurements

Prior to analysis, several pre-processing steps were carried out in ImageJ (FIJI) to improve the quality of images as required. A custom MATLAB script was written to analyse vessel diameter using the full width at half maximum.

In high-speed line scan experiments, similar methods were used to measure the diameter of capillaries imaged using sliding windows of 40 ms. Red blood cell velocity (RBCV) measurements were calculated with a radon transform using freely available code.^29^ A coefficient of variation was calculated for RBCV for each individual vessel to measure temporal variation, by dividing the standard deviation over time by the mean value. To calculate haematocrit, images acquired of shadows cast by RBCs were binarised and the percentage area taken up by RBCs was calculated (black pixels vs. white pixels).

### Neuronal fluorescence measurements

Regions of interest (ROIs) over individual neurons were identified in GCaMP6f recordings, using the freely available software Suite2p.^30^ A fluorescence signal was extracted from each ROI, along with a background measure (F_neu_). To reduce out-of-focus neuropil contamination of the signal, 0.7 × F_neu_ was subtracted from each ROI.^31^

### Baseline analysis (no visual stimulation)

Neuronal measures: Baseline measures of neuronal activity were computed in rest (no locomotion) epochs lasting at least 10 seconds by counting peaks per minute and the size of these peaks. During rest, the correlation of ROIs within a field of view was measured.

Haemodynamic measures: For data that were recorded in the absence of a visual stimulation, rest periods were found as described above for neuronal activity and an average value per vessel or animal (as specified in individual figure legends) was calculated for each measured parameter.

Detection of hippocampal vessel responses to local calcium events: Peaks in calcium traces were identified as being at least 1.5 times the standard deviation of the whole trace average, and 2 seconds apart from the nearest peak. The corresponding vessel traces (i.e. in the same field of view (FOV) as neuronal region of interest) were then cut around the same time points. Responsive diameter traces were those where a dilation greater than 1 times the standard deviation of the baseline occurred for more that 0.5 seconds within 5 seconds of the calcium event. Response thresholds were chosen to best separate responses from non-responses.

### Visual stimulation data analysis

Neuronal and vessel diameter data acquired from visual stimulation experiments in V1 were cut into trials around the stimulus presentations and averaged to yield a mean response per vessel or animal, as specified in figure legends. Only trials where there was no significant locomotion during the period two seconds prior to stimulation onset or during the stimulation period were used. Responses were then classified as responsive or non-responsive. A percentage response rate was also calculated per vessel or animal and for vascular recordings, data was weighted according to the number of contributing trials. In V1, neurovascular coupling indices (NVCindex) were calculated by dividing each vascular AUC by the average neuronal AUC for each genotype (as some mice did not express GCaMP6f in neurons, so calcium data was not available for each mouse). In CA1, where neuronal activity was measured local to the vasculature, the NVCindex was calculated by dividing responding vessel diameter peaks by the corresponding neuronal calcium peaks. Similarly in CA1, net haemodynamic peaks were divided by the corresponding CMRO_2_ peaks

### Power spectrum analysis

Welch’s power spectral density estimates were computed across all traces (including locomotion epochs) for arteriole diameters, calcium fluorescence traces and capillary diameter traces. All spectra were computed from data recorded in the absence of visual stimulation (except for capillary traces in V1 which were collected during visual stimulation, however the expected increase in power as a result is not expected to be near 0.1 Hz). All data was detrended by subtracting the baseline (the 8th percentile calculated over 15 second time windows^32^). Discrete Fourier transforms were then carried out across 60 second time windows using the inbuilt MATLAB function “pwelch”. Data below 1 Hz was selected and interpolated to the same length using linear interpolation. All data underwent outlier removal based on the value at 0.1 Hz (all values greater than 3 standard deviations above the mean were removed). This resulted in the removal of a total of three pial arteriole traces (1 × APOE3, 2 × APOE4), two capillary traces in V1 (1 × APOE3 and 1 × APOE4), three hippocampal vessel traces (1 × APOE3 and 2 × APOE4), and no calcium traces.

## Vascular density calculations

APOE-TR mice underwent transcardial perfusion under terminal anaesthesia as previously described.^9^ Vascular density was calculated from the length of vascular skeleton per unit volume, using the “Analyse Skeleton” plugin in FIJI/ImageJ.^33^

## Statistics

On all summary graphs, the distribution of data is indicated either by plotting all data points or violin plots where the number of points is large. Medians and interquartile rage on violin plots or means ± SEM are additionally shown where indicated and standard deviations are reported in appendix (i). Individual data points represent individual blood vessels, brain slices, imaging sessions or animals as specified in figure legends. Statistical analyses were conducted in GraphPad Prism 8, SPSS, RStudio or MATLAB. Where relevant, data whose residuals were normally distributed, as determined by a D'Agostino-Pearson and Shapiro-Wilk test, were compared using an unpaired t test, with a Welch’s correction applied if variances were unequal as determined by an F test. Data that were not normally distributed were compared using a non-parametric Mann-Whitney U test. Linear mixed models (LMM) were carried out using ‘lmer’ in RStudio, with random effects specified in appendix (i). For correlation analyses non-normal data were analysed using Spearman’s rank correlation. To weight response frequency data, so that vessels with a larger number of contributing trials contributed more to the mean, a weighted least squares linear regression was used and the mean was weighted by the number of contributing trials. P values below 0.05 were considered significant, and those below 0.1 as trending towards significance.

## Results

### Vascular density, baseline flow, and blood oxygenation are not different in APOE3-TR and APOE4-TR mice

**Figure 1.**
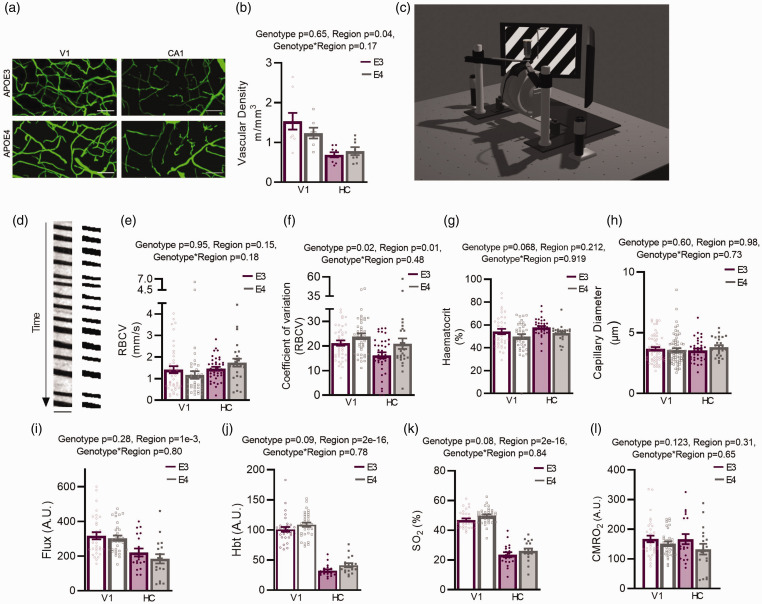
Under baseline conditions functional measurements are largely preserved in APOE4 mice within brain regions. (a) Example z-projected images of FITC-gelatin perfused vessels (green). Scale bars: 50 μm. Projected depth: 45.8 μm. (b) Vascular density (of vessels <7 μm in diameter) was reduced in HC compared to V1, but there were no effects of APOE genotype. (c) Schematic of in vivo imaging set up. Screens display a visual stimulation (drifting grating) as presented to animals with a V1 surgery. Net haemodynamic measurements were recorded using the Oxy-CBF probe and individual vessels and neurons were imaged using 2 photon microscopy. (d) Example output from a capillary line scan after preprocessing (left) and the same image binarised (right) for haematocrit measurements. Scale bar (y) = 214 ms, (x) = 4.8 μm. No region or genotype differences were found in RBCV (e) or in haematocrit (g) measures, but RBCV coefficient of variation (CV) was increased in APOE4 animals across regions, and decreased in HC compared to V1 (f). (h) No genotype differences were observed in capillary diameters. Regional differences in net measurements of (i) flux, (j) HbT and (k) SO_2_ were observed, with values being lower in HC vs V1. Trend level increases were observed in HbT and SO_2_ measures of APOE4 mice and (l) No genotype or regional differences were observed in net measurements of CMRO_2_. Individual dots on bar plots represent individual slices (B), individual vessels (E–H) and individual recording sessions (I–L). See appendix (i) for sample sizes and details on statistical tests.

Unlike in previous studies,^14,16^ we did not see a reduction in vascular density in 3–4 month APOE4- vs. APOE3-TR mice by imaging fixed brain tissue with the vasculature filled with a fluorescent gelatin (Figure 1(a) and (b)). We did observe a reduced vascular density in HC compared to V1.^9^

We then investigated baseline haemodynamic function (in the absence of locomotion or visual stimulation) using awake head-fixed mice with either a cranial window implanted over the visual cortex or CA1 (Figure 1(c)). Unlike previous literature,^14,16^ we observed minimal differences in resting haemodynamics between genotypes. Studying individual vessels using 2-photon microscopy, baseline RBC velocity (Figure 1(d) and (e)) and haematocrit (Figure 1(d) and (g)) did not differ between genotypes or brain region, nor did the diameters of individual capillaries (Figure 1(h)). The coefficient of variation (CV), reflecting temporal fluctuations in RBCV, was lower in HC than in V1 (Figure 1(f)), suggesting more homogenous flow. This may promote oxygen extraction ^34^ in hippocampal vessels, compensating for the lower hippocampal CBF. Conversely, flow was more variable in APOE4 than APOE3 vessels in both brain regions which may indicate less efficient oxygen extraction in APOE4 mice.

Macroscopic flow properties assessed using combined haemoglobin spectroscopy and laser doppler flowmetry revealed no differences between genotypes in RBC flux, total haemoglobin (HbT), oxygen saturation (SO_2_) or the cerebral metabolic rate of oxygen consumption (CMRO_2_) (Figure 1(i) to (l)). Consistent with our previous findings,^9^ flux, HbT and SO_2_ were lower in HC than V1, while CMRO_2_ was the same.

### Neuronal activity is unchanged in the hippocampus and visual cortex at baseline, but APOE4-TR animals display a hyperactive phenotype in response to visual stimulation

**Figure 2.**
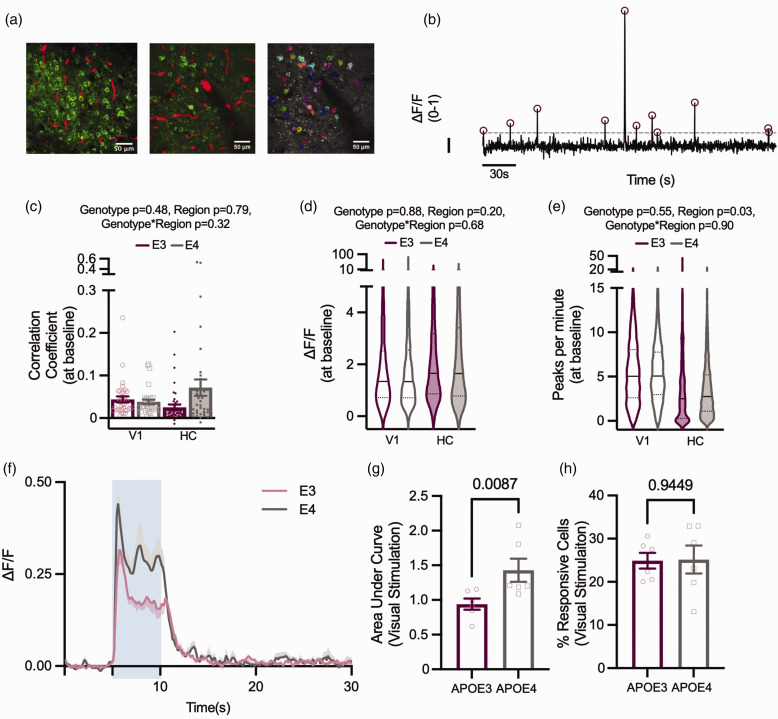
Under baseline conditions, genotypes display the same neuronal characteristics, but during visual stimulation APOE4 animals have larger neuronal responses. (a) Left: Thy1-GCaMP expressing neurons (green) and Texas Red filled vasculature (red) from CA1 (left panel) and visual cortex (middle panel). Right panel: ROIs overlying individual cells as detected using Suite2P (Pachitariu et al; 2016). (b) Example ΔF/F trace from one ROI, normalised between 0–1. Dashed line depicts threshold for peak selection, circles represent detected peaks and scale bar represents ΔF/F of 0.1 on the y axis and 30 s on the x axis. At baseline, (c) the correlation between individual cells within a recording, (d) the size of detected peaks per ROI, and (e) the number of detected peaks per ROI did not differ between genotypes. There were no regional differences in the correlation between cells or size of detected peaks, but the number of peaks per minute was lower in HC than V1. In visual cortex, the size of the neuronal response to 5 s visual stimulation (blue shaded region) was larger in APOE4 animals (f, g) but the percentage of neurons that responded to visual stimulation did not differ between genotypes (h). Individual data points plotted in barcharts represent individual fields of view (FOV, C) or animal averages (G,H) and violin plots represent values for individual cells.

To allow us to relate vascular function to neuronal activity – i.e. to study neurovascular coupling –we imaged neuronal calcium activity in V1 and CA1 pyramidal cells (Figure 2(a)) during baseline conditions, as well as evoked activity in the visual cortex during presentation of a drifting grating (Figure 1(c)). We assessed the number and size of individual calcium responses, how correlated cells’ firing was across the FOV and measured the network response across all cells during visual stimulation. At baseline, neuronal activity was no different between APOE3 and APOE4 mice in either region (correlation, size or frequency of calcium peaks; Figure 2(c) to (e))

In contrast, there was a larger change in fluorescence in response to a visual stimulation in APOE4-TR mice (Figure 2(f) and (g)), though the number of cells that significantly increased their calcium during visual stimulation was unchanged (Figure 2(h)). Therefore, calcium signals increase more in cells responsive to a visual stimulus in APOE4 compared to APOE3-TR mice.

This effect could be due to increased calcium signals to the same level of electrical excitation, or increased depolarisation or spiking of these cells during visual stimulation. Both options are plausible: calcium handling has been shown to be impaired in astrocytes of male APOE4-TR mice,^35^ while neurons of old APOE4-TR mice have been shown to be hyperactive due to decreased inhibitory GABAergic tone.^17^ We could not test whether a similar enhanced response to stimulation occurs in the HC as neurons are not activatable by a discrete stimulus in the same way as in V1.

### Vascular responses to visual stimulation are unimpaired in the capillary bed, but pial arterioles are less responsive in the neocortex of APOE4-TR mice

The ability of vessels to respond to increased neuronal activity might be compromised in APOE4 carriers as alterations in pericyte function increase BBB permeability,^14^ and APOE4 mice undergoing acute surgery have decreased functional hyperaemia.^16^ We used two separate approaches to study neurovascular coupling in HC and V1 because of different experimental constraints in the two regions. GCaMP6f expression was patchier in V1, so it was not always possible to record calcium signals next to imaged blood vessels, but we could track stimulus-evoked responses from the pial arterioles into the capillary bed. However, in HC, whilst we could not measure stimulus-evoked responses, and the large feeding vessels were too deep to be imaged, we could image capillaries and small arterioles adjacent to spontaneous local neuronal calcium signals.

**Figure 3.**
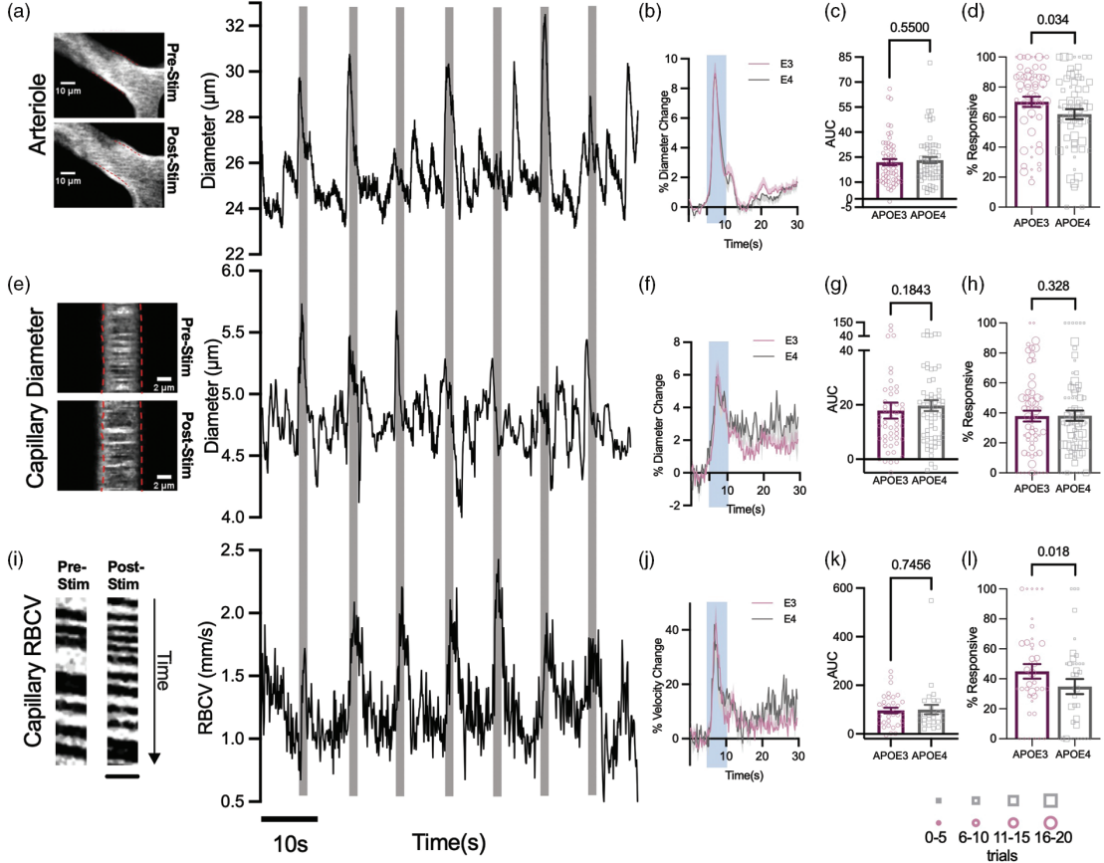
Vascular responses to visual stimulation are less reliable in visual cortex of APOE4 mice, but response sizes do not differ between genotypes. Example images and time series traces of pial arteriole diameter (a), capillary diameter (e) and capillary RBCV (i) responses to visual stimulation (Scale bar (y) = 260 ms, (x) = 5.6 μm). The response magnitude did not differ between genotypes in either the arteriole diameter (b, c), capillary diameter (f, g) or capillary RBCV (j, k). In APOE4 mice, the pial arteriole (d) and RBCV (l) response frequency was reduced, however there was no difference in the frequency of response to visual stimulation in capillary diameters (h). Response frequency was weighted according to the number of contributing trials per vessel. The size of the individual data points reflects the number of trials, as per legend. Individual dots on bar plots represent the data points for individual vessels. See appendix (i) for sample sizes and statistical tests.

In V1, we measured stimulus-evoked diameter changes in pial arterioles and downstream capillaries, and RBCV in capillaries. In a healthy system, visual stimulation evokes neuronal activity in the visual cortex and dilations and RBCV increase across vessels (Figure 3(e), (a) and (i)). The frequency and size of dilations in response to visual stimulation were the same in the capillary bed of APOE3 and APOE4-TR mice (Figure 3(a), (e) and (i)). However, the frequency of stimulus-evoked upstream pial dilations and percentage of RBCV increases was reduced in APOE4-TR mice (Figure 3(d) and (l)). The size of these responses, when they did occur, was no different between genotypes (Figure 3(b), (c), (j) and (k)), suggesting the potential for dilation remained the same in both genotypes. This was also the case when the size of the pial arteriole response was normalised to the larger neuronal responses in APOE4 vs APOE3-TR mice to generate a neurovascular coupling index (NVCindex; Supplementary Figure S1). Because vasodilation propagates upstream from the capillary bed,^36,37^ this decreased reliability of APOE4-TR mice pial vessels in the absence of changes in capillary responsiveness could be as a result of an impairment in the propagation of dilation from the capillaries, rather than in the capacity of pericytes and smooth muscle cells to dilate. Future work should be carried out to test this hypothesis.

However, these alterations in vascular responsivity did not affect haemodynamic responses, with CBF, SO_2_ and blood volume changes to visual stimulation the same across genotypes (Supplementary Figure S2). Neurovascular activity observable here at the single vessel level is therefore not sufficient to affect macroscopic measurements of functional hyperaemia. The lack of a genotype effect on overall CBF is consistent with the unimpaired capillary responses, as the capillary bed represents the majority of the resistance in the vascular bed, so is expected to mediate most of the increase in CBF,^38,39^ but is different from previous reports in anaesthetised mice showing a substantial reduction in functional hyperaemia.^16^

**Figure 4.**
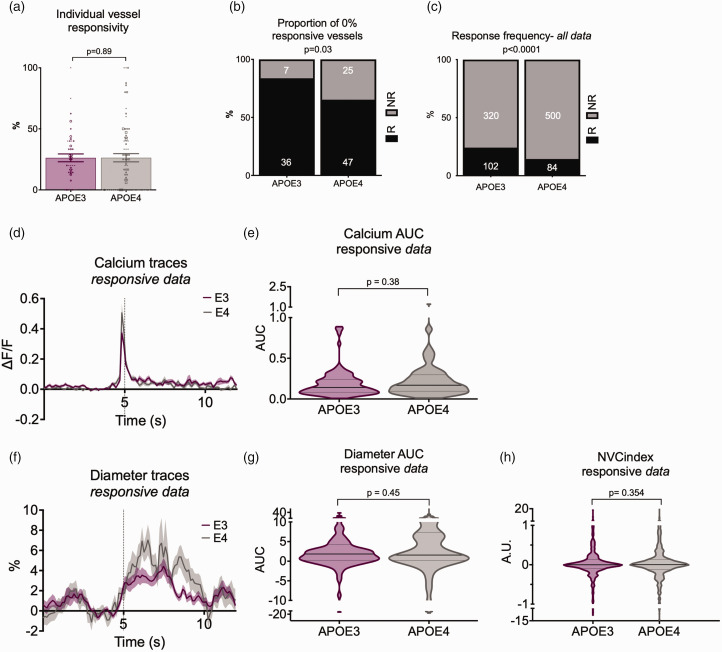
Vascular responses to preceding calcium events are less reliable in CA1 of APOE4 mice, but response sizes do not differ between genotypes. (a) Calcium evoked response frequency per individual vessel (for vessels with at least 3 contributing trials) was not different between APOE3 and APOE4 mice when data was weighted according to the number of contributing trials. The size of the individual data points reflects the number of trials, with the smallest dots corresponding with the data with the least trials (*symbol size 1: 3–5 trials, symbol size 2: 6–10 trials, symbol size 3: 11–15 trials, symbol size 4: 16+ trials*). (b) Local net calcium events were detected for each recorded vessel, and all recordings with >=3 calcium peaks were retained. Vessel responsiveness to the local net calcium event was classified, and overall response rates (%) were calculated per vessel. Significantly more vessels never responded (0% response rate) to preceding net calcium activity in APOE4 mice compared to APOE3 (c) Vessel response frequency to preceding individual calcium events was higher in APOE3 than APOE4 mice. (d) calcium peaks and (f) diameter traces were plotted for responsive dilations only (e, g) The AUC of the (e) preceding calcium peaks and vessel peaks (g) was not different between genotypes and (h) The neurovascular coupling index (dilation peak/calcium peak) was not different between genotypes. The individual values in the bar charts represent individual vessels and for D-H data points represent individual calcium events (and corresponding vascular responses). See appendix (i) for sample sizes and statistical tests.

In the HC, where we could relate local calcium changes to single vessel dilations, we found that while the average responsivity per vessel was not different across genotypes (Figure 4(a)), there were more blood vessels that never responded to local calcium events (Figure 4(b)), and fewer calcium events resulted in a dilation of local vessels in APOE4 vs APOE3 mice (Figure 4(c)), despite similar increases in local neuronal activity (Figure 4(d) and (e)). As in the visual cortex, dilations were not significantly different in size when they did occur (Figure 4(f) to (h)). The reduced reliability of vascular responses was not sufficient to reduce regional responses to net changes in neuronal activity, as assessed by the size of fluctuations in Hbt, blood flow or sO2 following fluctuations in CMRO2 (Supplementary Figure S3).

These results show that in both HC and V1, neurovascular coupling was mildly disrupted, as blood vessels dilated less frequently to increases in neuronal activity, but that these changes did not affect overall haemodynamics.

### APOE4-TR mice have impaired vasomotion

**Figure 5.**
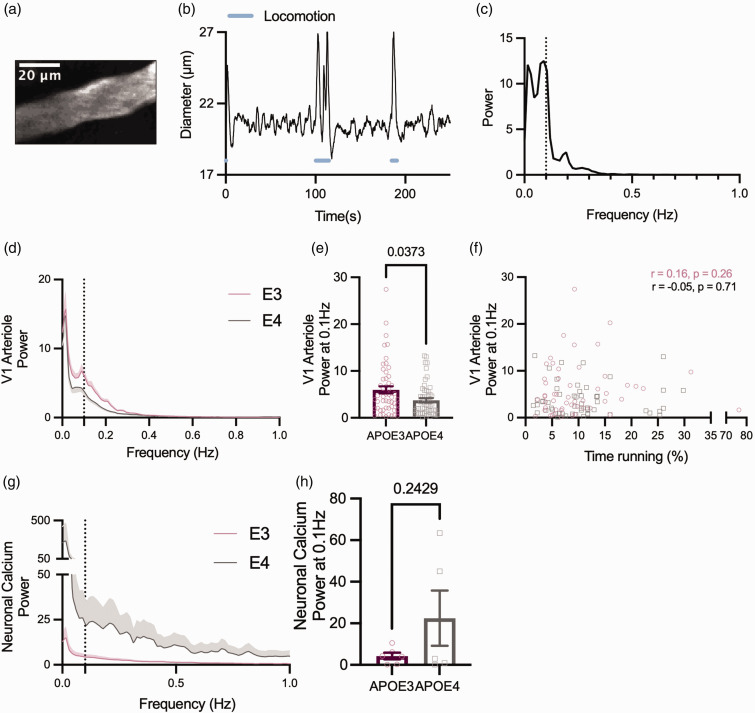
Vasomotion is reduced in the arterioles but not capillaries of APOE4 mice. (a) Example 2-photon image showing a representative pial arteriole from V1 (b) Example V1 pial arteriole diameter trace showing spontaneous fluctuations in diameter in the absence of locomotion (blue line) or sensory stimulation. (c) Power spectrum of diameter trace shown in B. Peak frequency can be observed at ∼0.1 Hz (dashed line). (d) Average power spectra of arteriole diameter traces from APOE3 (pink) and APOE4 (grey) mice. Dashed line at 0.1 Hz. (e) Arterioles from APOE4 mice have lower power at 0.1 Hz. (f) The power at 0.1 Hz was not affected by the amount of time spent running for either genotype. (g) Average power spectra for thy1-GCaMP6f fluorescence traces from V1 in APOE3 and APOE4 animals and (h) There was no observed difference in the power at 0.1 Hz for neuronal calcium. Individual data points represent values for individual vessels (E–F) or animal averages (H). See appendix (i) for sample sizes and statistical tests.

We tested whether vasomotion in pial arterioles in V1 (Figure 5(a)) was affected by APOE genotype. Fourier transforms of our diameter traces (Figure 5(b)) decomposed the time course of diameter changes into a spectrum of the power of fluctuations at different frequencies, revealing peaks of increased power at the vasomotion frequency (∼0.1 Hz) (Figure 5(c)). The power at this frequency was strikingly lower in APOE4 mice (Figure 5(d) and (e)).

It has been demonstrated by others that vasomotion, although an intrinsic vascular property, can be entrained by neuronal activity,^40^ in particular the gamma band envelope, so we tested whether the decrease in vasomotion was due to altered neuronal activity across the frequency domain. Fourier analyses on GCaMP6f data, though acquired at too low a frequency to directly measure gamma band activity, can reveal oscillatory activity that correlates with vasomotion.^41^ However, there was not only no peak in the neuronal power spectrum in the vasomotion range (Figure 5(g)), but the power also did not differ between genotypes (Figure 5(h)). That the APOE4-TR calcium power spectra, though variable, were not reduced at low frequencies relative to APOE3-TR signals, suggests that the observed reduction in oscillatory activity in APOE4 mice is likely vascular in origin, at least as far as can be reflected in excitatory neuronal calcium signals. There were no observed peaks nor significant differences in power across genotypes at the vasomotion frequency in capillary diameters in V1 nor in vessel diameters recorded in CA1 (where large feeding arterioles lie beyond our maximum imaging depth; Supplementary Figure S4), consistent with our previous observations of low vasomotion in these vessels.^42^

### Age does not modulate neurovascular alterations in APOE4 animals

**Figure 6.**
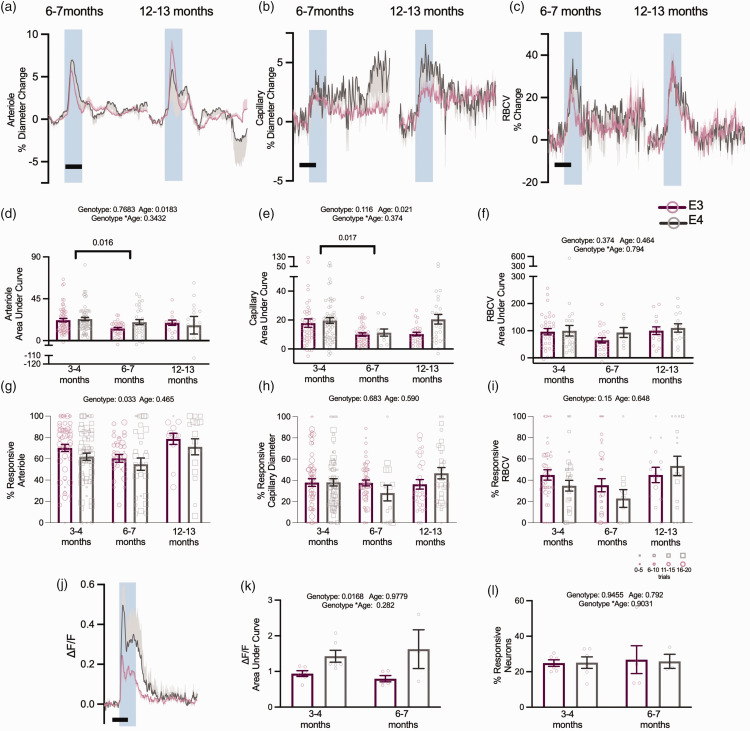
Age does not robustly modulate vascular or neuronal responses to visual stimulation. There was no genotype effect on the response magnitude to visual stimulation in animals across all age points (a–f). In both arteriole diameter and capillary diameter measurements, there was an effect of age, with 3–4 month animals displaying larger responses to stimulation than at 6–7 months, however this effect was not observed in 12–13 month old animals. Response frequency to visual stimulation continued to be modulated by genotype across all ages in arteriole responses to stimulation (g), however they were not affected by age. Neither capillary diameter response frequency (h) or RBCV response frequency (i) was modulated by age or genotype. At 6–7 months, neuronal responses to stimulation continued to be larger in APOE4 animals (j, k), but this effect was not modulated by age and The response frequency of neurons to stimulation was not affected by age or genotype (l). Individual dots on bar plots represent the values for single vessels (d–i) or animal average (k, l). Response frequency was weighted according to the number of contributing trials per vessel. The size of the individual data points reflects the number of trials, as per legend Scale bars = 5 s. Shaded regions represent the presentation of a visual stimulation. See appendix (i) for sample sizes and statistical tests.

**Figure 7.**
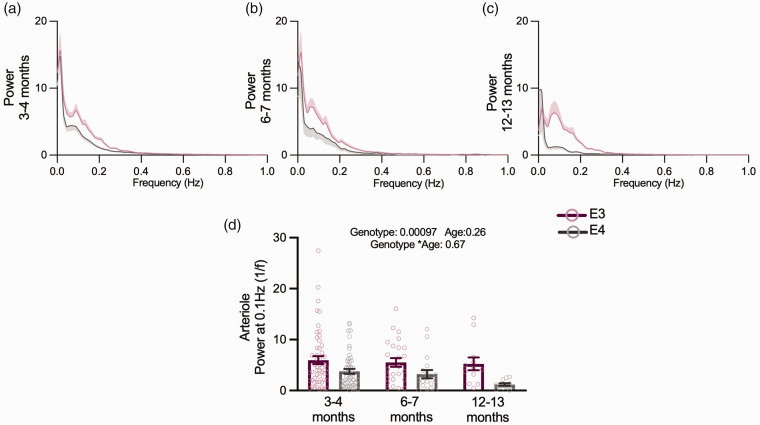
Vasomotion is reduced across all age points but does not appear to be modulated by age. Average power spectra of arteriole diameter traces across all three age points (a, b, c) and APOE4 animals had lower power at 0.1 Hz (d). Individual dots on bar plots represent the values for single vessels. See appendix (i) for sample sizes and statistical tests.

For a subset of experiments in the visual cortex we recorded from older mice, to investigate if age affected neurovascular function.^14^ Due to mice availability, we could only measure neuronal activity at 3–4 and 6–7 months, while vascular features were additionally measured at 12–13 months. The size of the dilations of capillaries and arterioles decreased across genotypes between 3–4 and 6–7 months, but no consistent age-related decreases were observed up to 12 months (Figure 6(a) to (f)). Where responses were affected by genotype in young animals, the same responses were generally seen in older animals, and no genotype effects emerged in any other metric. Specifically, the response frequency (Figure 6(g)) and vasomotion (Figure 7(a) to (d)) of pial arterioles remained significantly lower in APOE4 mice. Neuronal calcium during visual stimulation and baseline RBCV variability remained enhanced in APOE4-TR mice (Figure 6(j) and (k); Supplementary Figure S5). One measure – the frequency of RBCV increases to visual stimulation – was no longer different between APOE4- and APOE3-TR mice (Figure 6(i)) when studied across the whole age range studied, though not because of any significant differences between the ages studied.

Together, these data suggest that, rather than declining with age, neurovascular function in APOE3- and APOE4-TR mice is stable, showing specific and persistent differences in pial arteriole responsivity, irregular vasomotion and neuronal function consistently from a young age.

## Discussion

This was the first study to elucidate the effect of APOE genotype on neurovascular function at single neuron and vessel resolution in both the visual cortex and hippocampus of awake mice. Generally, we found milder effects of APOE4 genotype than has been previously suggested. We did not observe differences in vascular density, baseline CBF, blood SO_2_, or RBCV in single capillaries. Net increases in blood flow, sO_2_ or blood volume, or the size of single vessel dilations in response to visual stimulation were also not affected (unlike previously found for somatosensory stimulation^16^). Pial and hippocampal arterioles were, however, less likely to dilate to neuronal activation, and in the visual cortex showed decreased vasomotion in APOE4 compared to APOE3-TR mice. Furthermore, unlike previously reported overall reductions in neuronal activity,^14^ we found an increase in V1 neuronal calcium signals during visual stimulation. These genotype differences were unaffected by age (up to ∼13 months), suggesting they represent a stable state that does not, itself, cause progressive dysfunction. These effects may, instead, interact with other factors to place the system more at risk of dysfunction when external events do occur (e.g. older age, infection). Experiments in even older animals, at 18 or 24 months, would be of value in further disentangling the effect of such interacting factors.

### Differences with previous studies

Previous studies have observed a large decrease in the CBF response to whisker stimulation in APOE4-TR mice,^16^ a decrease in vascular density in APOE4 carriers, and a decrease in neuronal activity in response to hind limb stimulation measured with voltage dyes.^14^ Future experiments in somatosensory cortex could rule out a regional difference between somatosensory and visual cortex, however a more likely mechanism is the difference in experimental preparation. In our experiments, animals are imaged while awake and alert, having been allowed to recover from surgery for at least two weeks. The other studies utilised an acute preparation in which the mouse underwent cranial window surgery and was still anaesthetised when neuronal or haemodynamic recordings were undertaken. As human APOE4 carriers are more sensitive to anaesthesia than APOE3 carriers,^24^ and APOE4 is associated with an enhanced inflammatory phenotype,^43^ mouse models of disease may be more susceptible to cortical spreading depression after invasive surgery.^44^ Another likely contributing factor to our milder results is that following surgery, our mice had ad libitum access to an exercise wheel to avoid sensory and activity deprivation when singly housed post-surgery. Our experimental conditions therefore better recapitulate the human condition than many studies that do not include this enrichment, but likely reduce the impact of APOE4 genotype. Our preliminary data for a further study ^45^ suggest that removal of the exercise wheel decreases neurovascular coupling, as the presentation of a visual stimulation elicited smaller and less frequent vessel dilations in APOE4 sedentary mice compared to APOE3 mice or APOE4 active mice. Our current findings, showing an enhancement of neuronal activity and a slight disruption of vascular function, are a much closer approximation to the mild changes seen in young to middle-aged human APOE4 carriers ^46,47^ than previous extreme changes in vascular function, which may well be because our experimental conditions better recapitulate the health status of human APOE4 carriers. Overall, this pattern of results suggests APOE4 genotype causes mild neurovascular changes in healthy subjects but interacts with ongoing dysfunction or lifestyle risk factors to cause more severe dysfunction.

### Physiological impact of APOE4 on neurovascular function

We initially hypothesised that the mild changes we observed in young animals might progress into worse pathology with age. Our data suggest that this is not the case. The alterations in V1 neuronal and vascular function remained largely stable from 3 to 12–13 months of age. Instead, therefore, our data suggest that APOE4-TR mice are different from APOE3-TR mice in specific, subtle, but consistent ways. This parallels findings in humans of the existence of subtle differences between healthy APOE3 and 4 carriers (higher default mode network functional connectivity in APOE4s), but the lack of age-dependent changes.^47^ As mentioned above, our mice had access to a running wheel. Exercise has been shown to modulate neurovascular function in a number of conditions, including in a mouse model of AD,^48^ and future work should aim to elucidate the effect of exercise and environmental enrichment in this particular mouse model. Thus in our study, the genotype differences do not cause significant or progressive pathology in healthy, active individuals, but may cause APOE4 carriers to be more sensitive to triggers that can then cause progression into a pathophysiological state. Such a trigger could be acute surgery under anaesthesia, experimental interventions such as decreased cerebral perfusion,^16^ environmental changes such as exposure to infection, a sedentary lifestyle, extreme age or, as in the two-hit hypothesis of AD, factors that initiate the accumulation of Aβ.^49^

A key issue for future research is then to untangle how existing differences between APOE3 and 4 carriers interact with additional triggers to cause a change from a stable state to progressive pathology and neurodegeneration. Because cardiovascular risk factors potentiate the risk of someone carrying an APOE4 allele developing AD,^50^ vascular factors are likely involved in this switch, while neuronal changes that could potentiate production of Aβ are also relevant. Some such APOE4-mediated differences are already known, including increased BBB permeability and pericyte inflammation.^14,15^ Our results add three further features for consideration: increased neuronal calcium signals to stimulation, decreased vessel responsivity, and decreased pial vasomotion.

### Neuronal calcium

Before our study, increased neuronal calcium levels have been previously reported in cultured neurons exposed to ApoE4 both at rest and in response to stimulation (NMDA application,^51^ or mechanical injury,^52^ though in the latter, ApoE4 had no effect on resting calcium). Whether or not these observations and the increased signals we measure in vivo are due to increased neuronal depolarisation or altered calcium handling (as has been found in APOE4-positive astrocytes from male mice^35^) the ubiquity of the involvement of calcium in cellular processes means that these alterations are likely to be physiologically relevant. The increase in calcium, and its ability to modulate synaptic strengths could therefore potentially underlie the increased connectivity and gamma band oscillations observed in human APOE4 carriers.^47^ It will be of interest to observe how such circuit changes develop in mice, to determine whether this contributes to the decrease in GABAergic inhibitory tone and electrophysiological hyperactivity that develops in old age APOE4 mice.^17^ Of particular interest is how these alterations affect how neurons respond to Aβ. Neuronal hyperactivity increases in response to Aβ, but predominantly in cells that are already active.^53^ Therefore, increased neuronal activity caused by ApoE4 may magnify the hyperactivity caused when Aβ is produced, exacerbating its pathophysiological effects.

### Vascular responsiveness

In our experiments, stimulus evoked pial dilations and calcium dependent dilation of CA1 arterioles and capillaries occur less frequently in APOE4-TR mice. These alterations in pial responsiveness were not sufficient to reduce the overall regional increase in CBF, HbT or oxygen delivery but do indicate something is altered in the pial vasculature of these APOE4-TR mice. Because the capillary bed responses were unchanged in the visual cortex, and the pial and CA1 dilations when they did occur were also of the same size in APOE3 and APOE4-TR mice, the capacity of the smooth muscle cells and pericytes of APOE4-TR mice to dilate may not be impaired, but instead the ability of dilations to spread upstream from the capillary bed to trigger upstream dilations may be reduced. A future research question would be whether endothelial calcium signals are altered as well as those in neurons, as these signals are involved in propagation of vasodilation via activation of transient receptor potential ankyrin 1 channels (TRPA1^54^) as well as in activating nitric oxide production, which itself can modulate propagation of vasodilation.^55^

### Pial arteriole vasomotion

Possibly the most striking difference between APOE3 and APOE4-TR mice was the decrease in low frequency fluctuations of pial arteriole diameter in APOE4 mice – a phenomenon known as vasomotion. The mechanism underlying this reduced vasomotion is unclear, especially as we show that smooth muscle cells can dilate to neuronal activity with similar size responses to visual stimulation in APOE4 mice. Though vasomotion has been found to entrain to neuronal activity,^40^ we find no evidence of a reduced neuronal drive on vasomotion, though this should be confirmed with electrophysiological measurements of local field potentials, to directly examine the gamma band envelope thought to entrain vasomotion.

Whatever the underlying mechanism, our results suggest another way in which, in APOE4 carriers, a system with reduced vasomotion could be stable, but at risk of decline if Aβ production increases. Vasomotion is known to be important for perivascular clearance of waste molecules, including Aβ, from the brain.^3^ APOE4 carriers have an increased Aβ burden both in AD and in cerebral amyloid angiopathy, probably as a result of reduced clearance.^56^ Whilst there are several mechanisms that have been shown to contribute to this, including reduced clearance across the BBB ^57^ and reduced degradation,^58,59^ our data and others’ showing increased perivascular deposits of exogenously applied Aβ in APOE4 mice^60^ point towards reduced perivascular clearance, due to a decreased driving force from vasomotion, as an additional mechanism. Thus, reduced vasomotion may not matter for the normal physiological functioning of the brain until Aβ production increases and clearance fails, allowing Aβ to accumulate further and exacerbate vascular dysfunction.

We did not find age-related differences in vasomotion power in this study where the oldest mice studied were 12–13 months. However future studies investigating the effect of age on vasomotion power would benefit from the use of an even older cohort (e.g. >18 months) or larger sample sizes (our 12–13 month cohort included 24 vessels across 7 APOE3 and APOE4 mice, and so our power to detect differences between young APOE4 and old APOE4 mice at alpha = 0.05 was only moderate at 62%).

## Conclusion

Together our data point towards a subtle yet robust effect of APOE4 on the neurovascular system across the lifetime. They indicate APOE4-TR mice have increased neuronal calcium signals in response to sensory stimulation that may contribute to the altered network activity observed in humans and older mice, as well as specific deficits in vascular responsiveness and vasomotion that are not on their own sufficient to alter oxygen delivery or cerebral blood flow in healthy mice. However, the nature of these changes suggests that they may be important features that contribute to the decline of the previously stable system when an additional challenge is encountered, be that further vascular damage, infection or initiation of Aβ accumulation. Investigation of how such factors interact with APOE genotype should therefore be interrogated to understand how APOE4 genotype confers risk of developing AD.

## Supplemental Material

sj-pdf-1-jcb-10.1177_0271678X231172842 - Supplemental material for APOE4 expression confers a mild, persistent reduction in neurovascular function in the visual cortex and hippocampus of awake mice Click here for additional data file. Supplemental material, sj-pdf-1-jcb-10.1177_0271678X231172842 for APOE4 expression confers a mild, persistent reduction in neurovascular function in the visual cortex and hippocampus of awake mice by Orla Bonnar, Kira Shaw, Silvia Anderle, Dori M Grijseels, Devin Clarke, Laura Bell, Sarah L King and Catherine N Hall in Journal of Cerebral Blood Flow & Metabolism
