# Supplementary material for: APOE4 expression confers a mild, persistent reduction in neurovascular function in the visual cortex and hippocampus of awake mice
Source: J Cereb Blood Flow Metab. 2023 Jun 23;43(11):1826–41. doi: 10.1177/0271678X231172842 (PMC10676141; doi:10.1177/0271678X231172842)
Supplement: sj-pdf-1-jcb-10.1177_0271678X231172842 - Supplemental material for APOE4 expression confers a mild, persistent reduction in neurovascular function in the visual cortex and hippocampus of awake mice [file sj-pdf-1-jcb-10.1177_0271678X231172842.pdf]

Supplementary Materials and Methods

Surgical Preparation

Animals were anaesthetised with isoflurane (~4% at induction, ~1.5 – 2% maintenance) and body temperature was maintained at 37°C using a homeothermic monitoring system (PhysioSuite, Kent Scientific Corporation). A 3 mm hole was created over the visual cortex or CA1, the dura removed, and for V1 surgery a glass window used to seal the exposed area. For CA1 surgery, ~1.3mm of cortex was aspirated and a custom-made cannula with 3mm glass coverslip was inserted into the craniotomy and then secured in place at the skull surface. Custom-made titanium head bars were fixed to the exposed skull to enable later head fixation. Animals were administered subcutaneous injections of saline 0.9% (400μL), buprenorphine (1.2μg, Vetergesic, Ceva), meloxicam (6.2 μg, Metacam, Boehringer Ingelheim) and dexamethasone (120 μg, Dexadreson, MSD Animal Health) at induction, and continued to receive meloxicam (100 μg) in food for three days post-surgery.

2-photon microscopy

In visual cortex, pial arterioles were identified by the presence of smooth muscle cells (in NG2-DSred animals), or by their morphology and orientation relative to the large pial veins, as well as their response to visual stimulation. Pial arterioles were then followed with x-y recordings (average pixel size: 0.42 x 0.42 μm^2, imaging speed range 3.8–7.6Hz) made after bifurcations, until the vessels penetrated the parenchyma and therefore could no longer be classed as pial. Penetrating arterioles were followed and branching capillaries were imaged using high speed line scans, to record both diameter and RBCV (average pixel size: 0.19 μm, average number of traversals/second = 1224). In animals positive for GCaMP6f, additional x-y recordings (average pixel size: 1.28 x 1.28 μm^2, imaging speed range 3.8–7.6Hz) were taken from the area proximate to vessel recordings

at a depth that allowed for clear visualisation of neuronal cell bodies (layer 2/3 (~z = -150μm)). In CA1, images were taken up to ~500 μm from the surface of the window. Both x-y recordings of neuronal calcium across a large FOV (256 × 256 pixels, speed range 6.10–15.26 Hz, speed average 7.75 Hz, pixel size average 1.80 μm) and smaller FOV recordings allowing for concurrent vascular and local neuronal calcium signals (256 × 256 pixels, speed range 3.05–7.63 Hz, speed average 6.64 Hz; pixel size average 0.23 μm) were obtained. RBCV measurements were obtained as above using high speed linescans (average pixel size: 0.18 μm, average number of traversals/second = 791).

Data analysis

Preprocessing of images included registration, despeckling and/or median 3D filtering. In addition, all images had the 'stack contrast adjustment' plug-in applied to minimise any light artifacts arising from the visual stimulation. A custom MATLAB script was used to extract vessel diameter. Briefly, a skeleton was generated along the centre of each vessel and an intensity profile was plotted along a line perpendicular to the vessel. Values were obtained across a window of five vessel skeleton pixels and averaged to obtain a mean value per window. This was repeated at every second skeleton point, allowing for diameter measurements to be made along the full vascular arbour. These measurements were then averaged along the vessel, resulting in an average diameter per frame.

RBCV measurements

In brief, the angle of shadows cast by the RBCs (that do not uptake fluorescent dextran) were measured and used to calculate the velocity across 40ms time windows that overlapped by 10 ms. Angles that were extremely large or small (because of motion artefacts) were removed. To further account for noise in the data obtained from line scans, traces went through either one (diameter) or two (RBCV) iteration(s) of outlier removal. In addition, for stimulation experiments, trials missing greater than 10% of data were excluded and those that remained were subsequently smoothed using a loess smoothing method (range: 1-5% span of the total number of data points, depending on which best represented the shape of the data). To determine AUC measurements, data was interpolated to fill in missing values, using a moving mean average.

Calcium measurements

To find peaks in the calcium signal, traces were first scaled so that all values fell between zero and one (equation ii). Peaks during rest periods were identified as being at least twice the standard deviation of the whole trace, and 0.25 seconds apart from the next peak. Each original trace was then normalised as is standard, (equation iii) and the size of each peak was determined and the number of peaks per minute was computed. During rest, the correlation of ROIs within a field of view was measured.

(ii) $\Delta F/F = (F-F_{min})/(F_{max} - F_{min})$

(iii) $\Delta F/F = (F-F_0)/abs(F_0)$

F represents the fluorescence trace. F represents the data trace (fluorescence), and F_0 represents the baseline period to which the rest of the data is normalised.

Visual stimulation data analysis

Significant locomotion was defined as an event that was more than one third of a second in length and/or less than one second apart from adjacent locomotion epochs.

Data were normalised to the 5 s baseline preceding the onset of visual stimulation using equation (iii). To determine if there was a response to stimulation, a threshold was set as twice the standard deviation of this 5 s baseline period. Any response larger than this threshold was deemed 'responsive'. For neuronal data all trials were averaged per cell and if the mean response was larger than the threshold, it was deemed a responsive ROI and subsequently averaged to provide a mean response size per animal.

For vascular data, these trials were averaged per vessel to provide a 'responsive only' trace. Each vessel had a different number of contributing trials (because mice ran different amounts for each recording) and those with a low number of trials were likely to provide more extreme response frequency values (e.g., 0% or 100%). Therefore, the number of contributing trials per vessel was used to weight data when calculating response frequency. When calculating the size of responses, area under the curve (AUC) measurements were taken during the stimulation period using the inbuilt MATLAB function "trapz" which utilizes trapezoidal numerical integration.

Vascular density calculations

Animals were first perfused with 0.1 M phosphate buffered saline (PBS) then 4% paraformaldehyde (PFA) in PBS, and were then perfused with 5% gelatin containing 0.2% FITC-conjugated albumin (at 37 °C). Following at least 30 minutes on ice, brain tissue was then extracted and stored in 4% PFA at 4˚C for 24 hours before being transferred to 30% sucrose in PBS for at

least 3 days. Tissue was then sliced at 200µm on a vibratome, and slices were imaged using confocal microscopy (Leica SP8, pixel size: 0.45 – 0.57µm).

Sample Size Considerations

To estimate the power of our findings, we have calculated the power post hoc. For our main findings of stimulus-induced calcium change (Figure 2) and vessel responsiveness (Figure 3) we have calculated the effect size (by taking the difference between group means and dividing by the standard deviation of the E3 group, as per Cohen (1988)), and used both effect and sample sizes to calculate power post-hoc. Our effect size for stimulus-induced calcium responses was 2.47 (nE3=6, nE4=6), resulting in >96% power for detecting differences between APOE groups at alpha = 0.05. Our effect size for vessel responsiveness in arterioles was 0.33 (nE3=33, nE4=37) and capillaries 0.32 (nE3=60, nE4=64), meaning our power to detect differences between APOE groups at alpha = 0.05 was low at 28% for arterioles and 40% for capillaries. However, the design of the whole study includes multiple replications of the key findings at different age points, and with consistent results being found across brain regions, mitigating this low power. In fact, huge sample sizes would be needed (of >155 capillaries & >160 arterioles per APOE group) to get to a power of 80%, and this would be unfeasible (>630 total vessels) and require 5x as many animals to undergo invasive surgery. Our animal numbers (6 animals per APOE group) are within the range recommended using the resource equation approach, Arifin & Zahiruddin, 2017; *sample size* = DF/*group size* + 1, where the aim is to have between 10 and 20 degrees of freedom).

Supplementary Figures

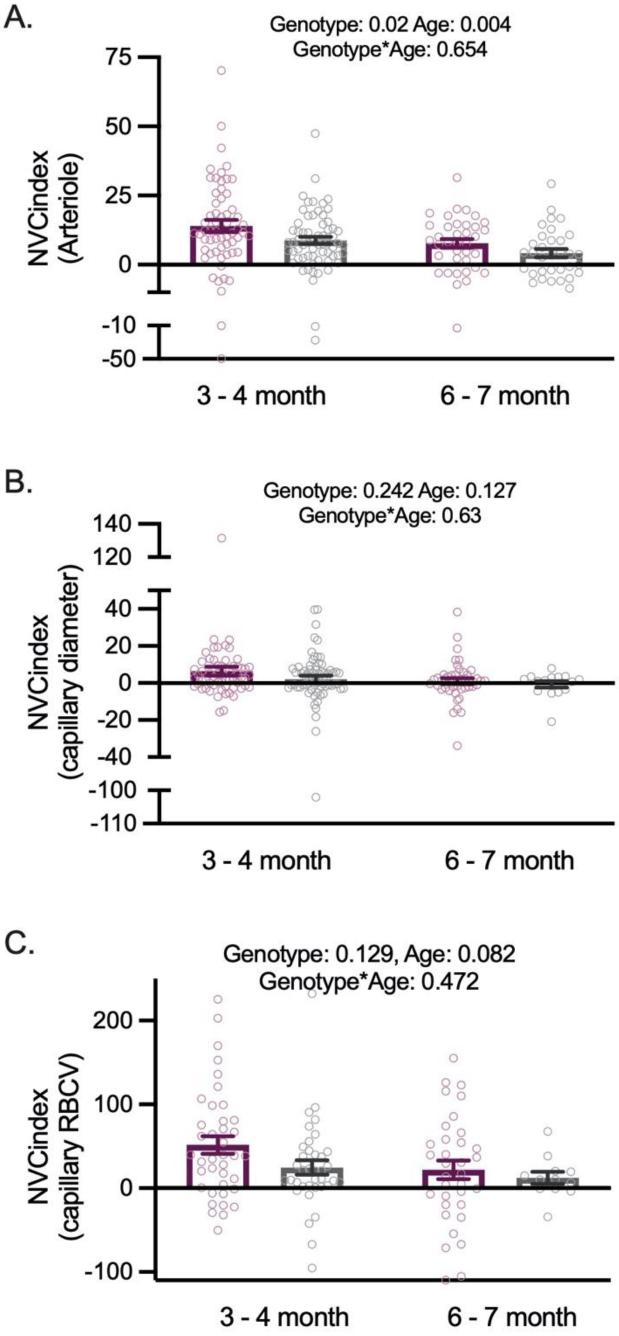

Supplementary Figure S1: Vascular response to visual stimulation normalised to average neuronal response to produce a neurovascular coupling index (NVCindexndex). Raised NVCindexndex in APOE3 animals was observed in pial arteriole **(A)** responses to visual stimulation**.** No difference was seen in capillary diameter **(B)** or RBCV NVCindex **(C).** Individual data points represent values from individual vessels. See appendix (i) for sample sizes and statistical tests.

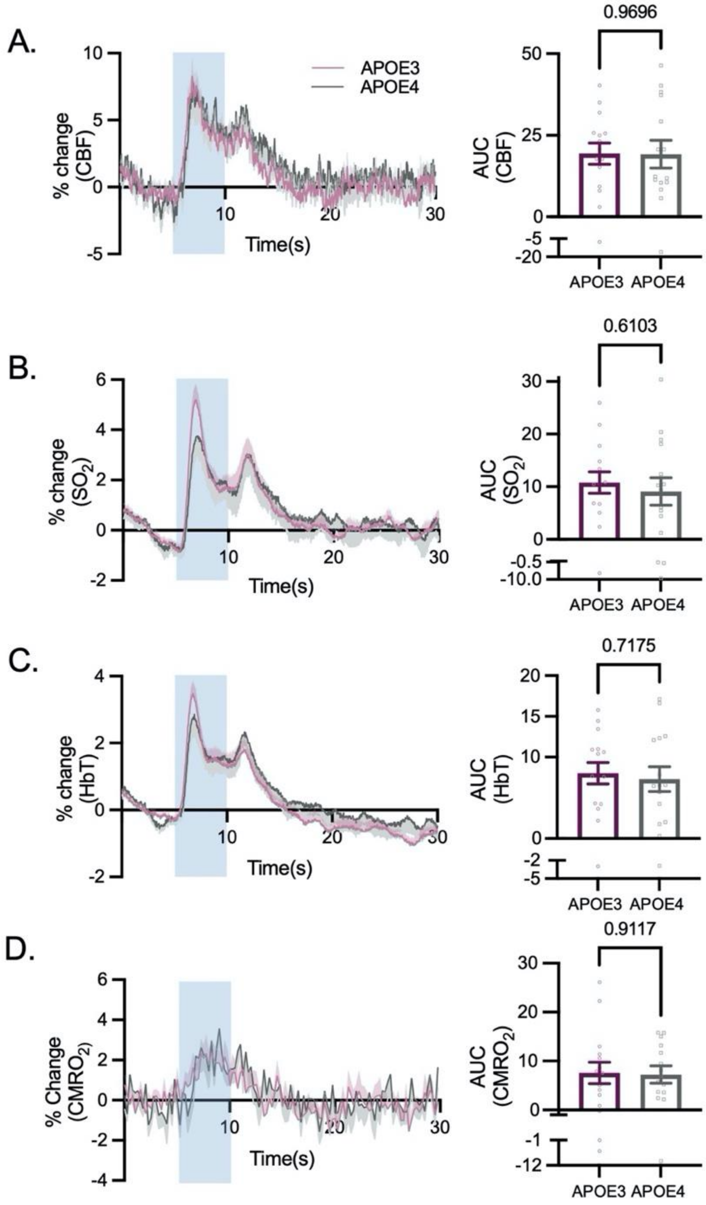

Supplementary figure S2. No effect of genotype was observed in net stimulus-dependent responses: Left panel shows average changes in net CBF **(A)**, sO_2 **(B)** total haemoglobin (HbT) **C)** and cerebral metabolice rate of oxygen consumption ($CMRO_2$); **D)** during 5s visual stimulation (blue shaded region). Right panel shows AUC for each during visual stimulation. Individual data points represent animal averages. See appendix (i) for sample sizes and statistical tests

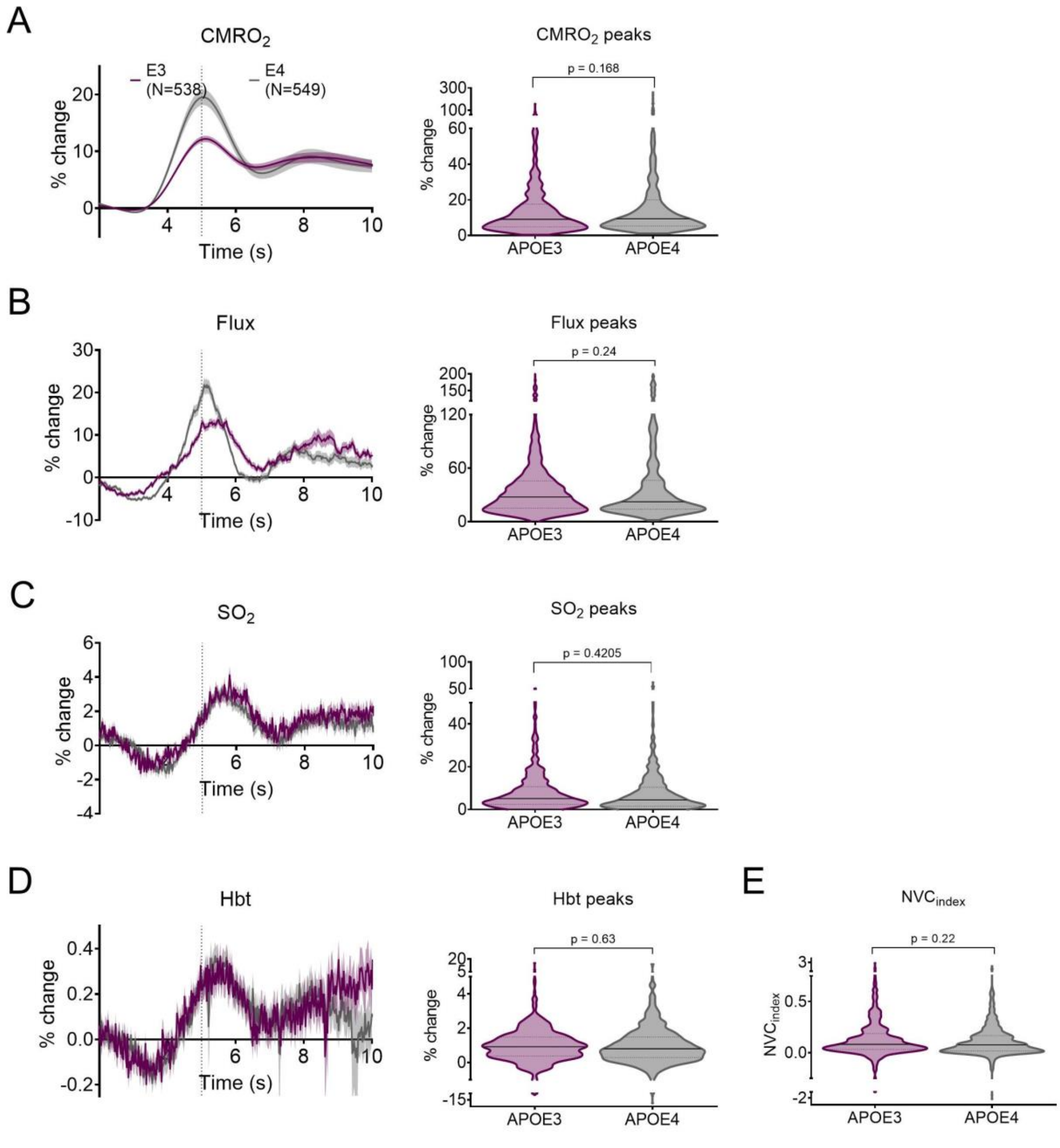

Supplementary Figure S3*: CMRO2-dependent haemodynamic responses in CA1*. **(A)** Detected peaks in CA1 Oxy-CBF probe recordings of net $CMRO_2$ were not larger in APOE4 than APOE3 mice, Corresponding CMRO2-dependent haemodynamic traces were then visualised for **(B)** flux, **(C)** oxygen saturation (SO_2) and **(D)** total haemoglobin (HbT). There were no significant genotype differences in CMRO2-dependent flux, SO_2 or HbT, **(E)** The

NVCindexndex was calculated by dividing HbT/$CMRO_2$, and showed no effect of genotype. Violin plots are composed of data points from individual haemodynamic events. See appendix (i) for sample sizes and statistical tests.

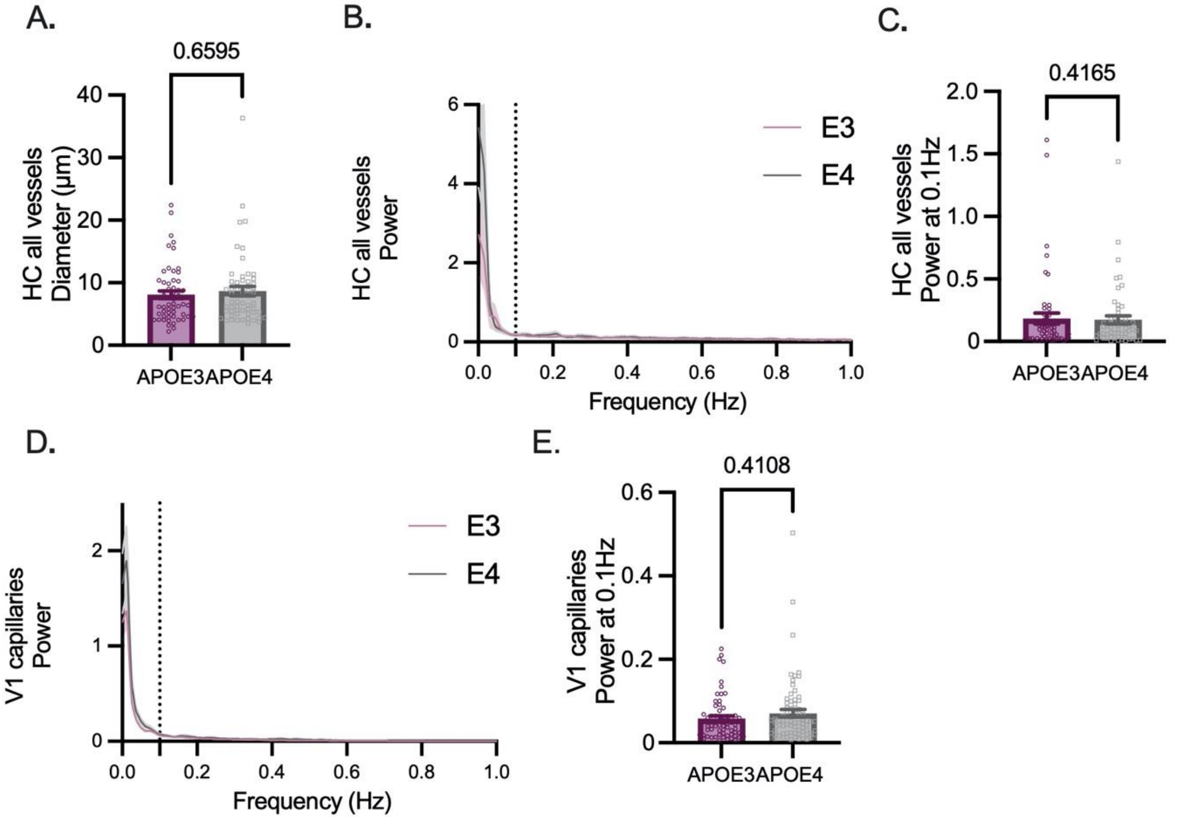

Supplementary Figure S4: In APOE3 and APOE4 mice, power at the vasomotion frequency is the same in the vessels of the hippocampus and in the capillaries in V1. The diameter of **(A)** all the vessels recorded in CA1 were not significantly different between genotypes. **(B)** Average power spectra of CA1 diameter traces from APOE3 (pink) and APOE4 (grey) mice for all recorded vessels. Dashed line at 0.1Hz. **(C)** Power at 0.1Hz did not differ between genotypes. **(D)** Average power spectra of V1 capillary diameter traces from APOE3 (pink) and APOE4 (grey) mice. **(E)** Power at 0.1Hz did not differ by genotype. See appendix (i) for sample sizes and statistical tests.

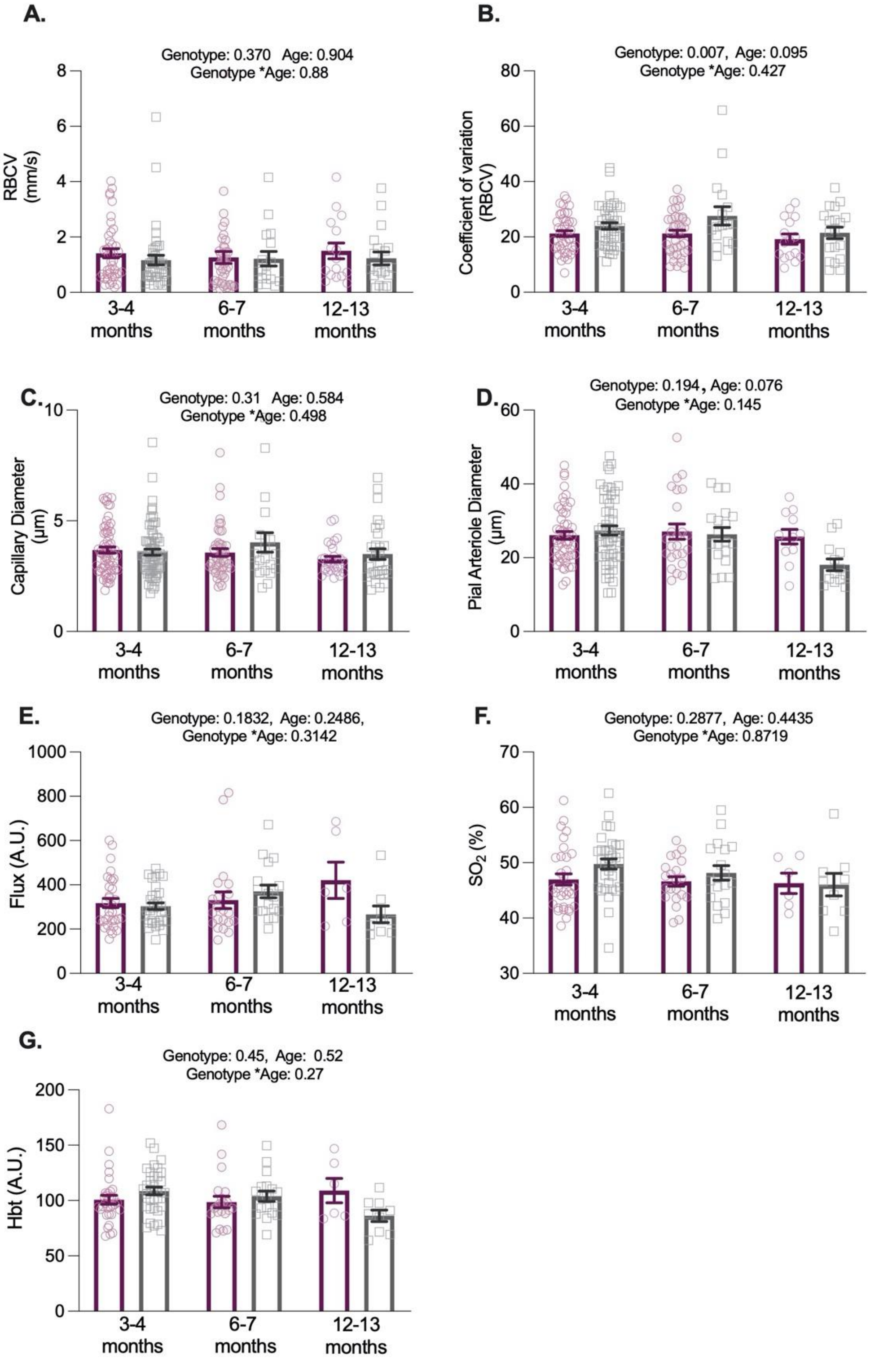

Supplementary Figure S5: ***Under baseline conditions functional measurements are largely preserved in APOE4 mice within brain regions. This effect is preserved in older animals.*** There was no effect of genotype or age on **(A)**

RBCV, **(C)** capillary diameter, **(D)** arteriole diameter, **(E)** flux, **(F)** SO_2 or **(G)** HbT. However a significant effect of genotype was observed in RBCV CV measurements (B). Individual data points on bar graphs represent single vessels (A-D); or recording sessions (E-G). See appendix (i) for sample sizes and statistical tests.
